# Perinatal Outcomes after Fetal Endoscopic Tracheal Occlusion for Isolated Congenital Diaphragmatic Hernia: Rapid Review

**DOI:** 10.1055/s-0041-1740596

**Published:** 2022-01-29

**Authors:** Juliana da-Costa-Santos, João Renato Bennini

**Affiliations:** 1 Department of Obstetrics and Gynecology, Universidade Estadual de Campinas, Campinas, SP, Brazil

**Keywords:** congenital diaphragmatic hernias, ultrasound diagnosis, prenatal ultrasonography, prognosis, systematic review, hérnias diafragmáticas congênitas, ultrassonografia, ultrassonografia pré-natal, prognóstico, revisão sistemática

## Abstract

**Objective** To compare the perinatal outcomes of fetuses with isolated congenital diaphragmatic hernia after fetal endoscopic tracheal occlusion (FETO) and antenatal expectant management.

**Data sources** In this rapid review, searches were conducted in the MEDLINE, PMC, EMBASE and CENTRAL databases between August 10th and September 4th, 2020. Randomized controlled trials (RCTs), quasi-RCTs or cluster-RCTs published in English in the past ten years were included.

**Study selection** We retrieved 203 publications; 180 studies were screened by abstract. Full-text selection was performed for eight studies, and 1 single center RCT met the inclusion criteria (41 randomized women; 20 in the FETO group, and 21 in the control group).

**Data collection** Data collection was performed independently, by both authors, in two steps (title and abstract and full-text reading).

**Data synthesis** There were no cases of maternal mortality. The mean gestational age at delivery was of 35.6 ±  2.4 weeks in the intervention group, and of 37.4 ±  1.9 weeks among the controls ( *p* < 0.01). Survival until 6 months of age was reported in 50% of the intervention group, and in 5.8% of the controls ( *p* < 0.01; relative risk: 10.5; 95% confidence interval [95%CI]: 1.5–74.7). Severe postnatal pulmonary hypertension was found in 50% of the infants in the intervention group, and in 85.7% of controls ( *p* = 0.02; relative risk: 0.6; 95%CI: 0.4–0.9). An analysis of the study indicated some concerns of risk of bias. The quality of evidence was considered moderate to low.

**Conclusion** Current evidence is limited but suggests that FETO may be an effective intervention to improve perinatal outcomes.

## Resumo

**Objetivo** Comparar os resultados perinatais de fetos com hérnia diafragmática congênita após oclusão traqueal endoscópica fetal (OTEF) e conduta expectante pré-natal.

**Fontes dos dados** Nesta revisão rápida, pesquisas foram conduzidas nas bases de dados MEDLINE, PMC, EMBASE e CENTRAL entre 10 de agosto de 2020 e 4 de setembro de 2020. Ensaios clínicos randomizados (ECRs), quase-ECRs e ECRs em *cluster* publicados em inglês nos últimos dez anos foram incluídos.

**Seleção dos estudos** Foram recuperadas 203 publicações; 180 destas foram triadas pelo resumo. Fez-se a leitura do texto completo de 8 estudos, e 1 ECR cumpriu os critérios de inclusão (41 mulheres aleatorizadas; 20 no grupo OTEF e 21 no grupo de controle).

**Coleta de dados** A coleta de dados realizada independentemente pelos dois autores, em duas etapas (título e resumo, e leitura do texto completo).

**Síntese dos dados** Não houve casos de morte materna. A idade gestacional média no parto foi de 35,6 ±  2,4 semanas no grupo de intervenção, e de 37,4 ±  1,9 semanas entre os controles ( *p* < 0,01). A sobrevida até 6 meses de idade foi relatada em 50% do grupo de intervenção, e em 5,8% dos controles ( *p* < 0,01; risco relativo: 10,5; intervalo de confiança de 95% [IC95%]: 1,5–74,7). Hipertensão pulmonar grave ocorreu em 50% dos lactentes do grupo de intervenção, e em 85,7% dos controles ( *p* = 0.02; risco relativo: 0,6; IC95%: 0,4–0,9). Uma análise do estudo indicou algumas preocupações quanto ao risco de viés. A qualidade da evidência foi considerada de moderada a baixa.

**Conclusão** As evidências atuais são limitadas, mas sugerem que a OTEF pode ser uma intervenção eficaz para melhorar resultados perinatais.

## Introduction

Congenital diaphragmatic hernia (CDH) is the failure in the closure of the pleuroperitoneal folds, which usually occurs between the fourth and tenth weeks of gestation. 1 2 3 4 5 This leads to herniation of abdominal organs to the thorax, which impairs bronchial ramification, reduces lung volume and the production of surfactant, and induces anatomic and functional adaptations in the pulmonary vasculature. Furthermore, herniated structures may result in mediastinal shift and associated hypoplasia of the cardiac structures ipsilateral to the hernia. 1 5

The estimated prevalence of CDH is of 1 to 4 cases in every 10 thousand live births, 1 2 but it may be higher if stillbirths and pregnancy terminations are considered, because many cases are associated with potentially-lethal syndromes.

The diagnosis is usually suspected prenatally, in the second trimester scan, which evidences abdominal organs inside the fetal thorax. 1 3 5 The sensitivity depends on the presence of associated anomalies, the size of the defect, the gestational age, and the sonologist's experience. 1 When other anomalies are detected, an association with genetic or chromosomic syndromes, such as trisomy 18, Pallister-Killian and Fryns syndromes, is possible. 5 The condition is then classified regarding prognosis using the lung-to-head ratio (LHR), defined as the relationship between the contralateral lung area and fetal head circumference (lower ratios indicate more serious cases), the observed/expected LHR (o/e LHR) and liver herniation. 5 6 In addition, serial scans may detect other characteristics of poor prognosis, such as fetal growth restriction and abnormalities in the amniotic fluid index. 1 2 3

For severe cases, the fetal endoscopic tracheal occlusion (FETO) procedure aims to prevent pulmonary hypoplasia and enable lung growth, which could reduce perinatal morbidity and mortality. 5 The FETO is a percutaneous procedure involving the placement of a balloon inside the fetal trachea, which retains lung fluid, elevating intrapulmonary pressure and enlarging the volume of the fetal lung. Traditionally, this intervention is performed in severe cases between 26 and 28 weeks of gestation and reversed around 34 weeks of gestation. Pregnancy continuation until term is expected to enable maturation of type-II pneumocytes and the production of surfactant. 1 There is recent evidence 5 7 indicating that this procedure could improve perinatal morbidity and mortality. Thus, the present study aimed to review and describe the quality of the evidence regarding perinatal outcomes after FETO, and compare it with the expectant management, for fetuses with isolated CDH.

## Methods

### Study Design

This rapid review was conducted according to the Cochrane Rapid Reviews Methods. 8 The risk of bias was assessed with the Risk of Bias 2(RoB 2) tool (Cochrane, London, UK). 9 The quality of the evidence was assessed using the Grading of Recommendation, Assessment, Development and Evaluation (GRADE) guidelines. 10 11 12 13 14 The final report was developed according to the Preferred Reporting Items for Systematic Reviews and Meta-Analyses (PRISMA) statement. 15 The study protocol is registered with PROSPERO CRD42020186509, which is available at https://www.crd.york.ac.uk/prospero/display_record.php?ID=CRD42020186509 .

### Search Strategy

The objective of the present study was to identify randomized controlled trials (RCTs), quasi-RCTs and cluster-RCTs comparing perinatal and maternal morbidities and mortality of pregnant women whose fetuses with isolated CDH underwent FETO or no intrauterine intervention. Searches were conducted on the MEDLINE, PMC, EMBASE and CENTRAL databases, using the terms related to CDH and fetoscopy, by an information specialist and the authors between August 10th and September 4th, 2020. Articles published in English in the past ten years were included for further selection. Unpublished studies and other types of publication were not sought. The searches were rerun prior to the final analysis.

### MEDLINE Search Strategy

The search strategy on the MEDLINE database was as follows: (((Hernias, Diaphragmatic, Congenital[MeSH Terms]) OR (“Hernias, Diaphragmatic, Congenital”[Title/Abstract] OR “Unilateral Agenesis of Diaphragm”[Title/Abstract] OR “Diaphragm Unilateral Ageneses”[Title/Abstract] OR “Diaphragm Unilateral Agenesis”[Title/Abstract] OR “Congenital Diaphragmatic Hernias”[Title/Abstract] OR “Congenital Diaphragmatic Hernia”[Title/Abstract] OR “Diaphragmatic Hernia, Congenital”[Title/Abstract] OR “Diaphragmatic Hernias, Congenital”[Title/Abstract] OR “Hernia, Congenital Diaphragmatic”[Title/Abstract] OR “Hernias, Congenital Diaphragmatic”[Title/Abstract] OR “Agenesis of Hemidiaphragm”[Title/Abstract] OR “Hemidiaphragm Ageneses”[Title/Abstract] OR “Hemidiaphragm Agenesis”[Title/Abstract] OR “Congenital Diaphragmatic Defect”[Title/Abstract] OR “Congenital Diaphragmatic Defects”[Title/Abstract] OR “Defect, Congenital Diaphragmatic”[Title/Abstract] OR “Defects, Congenital Diaphragmatic”[Title/Abstract] OR “Diaphragmatic Defect, Congenital”[Title/Abstract] OR “Diaphragmatic Defects, Congenital”[Title/Abstract] OR “Bochdalek Hernias” OR”Hernias, Bochdalek”[Title/Abstract] OR “Morgagni Hernias”[Title/Abstract] OR “Hernias, Morgagni”[Title/Abstract] OR “Morgagni's Hernias”[Title/Abstract] OR “Hernias, Morgagni's”[Title/Abstract] OR “Morgagnis Hernias”[Title/Abstract])) OR ((Hernia, Diaphragmatic[MeSH Terms]) OR (“Hernia, Diaphragmatic”[Title/Abstract] OR “Diaphragmatic Hernias”[Title/Abstract] OR “Hernias, Diaphragmatic”[Title/Abstract] OR “Diaphragmatic Hernia”[Title/Abstract]))) AND ((Fetoscopy[MeSH Terms]) OR (Fetoscopy[Title/Abstract] OR Fetoscopies[Title/Abstract] OR Amnioscopy[Title/Abstract] OR Amnioscopies[Title/Abstract] OR “Fetoscopic Surgical Procedures”[Title/Abstract] OR “Fetoscopic Surgical Procedure”[Title/Abstract] OR “Procedure, Fetoscopic Surgical”[Title/Abstract] OR “Procedures, Fetoscopic Surgical”[Title/Abstract] OR “Surgical Procedure, Fetoscopic”[Title/Abstract] OR “Surgery, Fetoscopic”[Title/Abstract] OR “Surgical Procedures, Fetoscopic”[Title/Abstract] OR “Fetoscopic Surgery”[Title/Abstract] OR “Fetoscopic Surgeries”[Title/Abstract] OR “Surgeries, Fetoscopic”[Title/Abstract] OR Embryoscopy[Title/Abstract] OR Embryoscopies[Title/Abstract] OR “Amnioscopic Surgical Procedures”[Title/Abstract] OR “Amnioscopic Surgical Procedure”[Title/Abstract] OR “Procedure, Amnioscopic Surgical”[Title/Abstract] OR “Procedures, Amnioscopic Surgical”[Title/Abstract] OR “Surgical Procedure, Amnioscopic”[Title/Abstract] OR “Surgery, Amnioscopic”[Title/Abstract] OR “Surgical Procedures, Amnioscopic”[Title/Abstract] OR “Amnioscopic Surgery”[Title/Abstract] OR “Amnioscopic Surgeries”[Title/Abstract] OR “Surgeries, Amnioscopic”[Title/Abstract] OR “Embryoscopic Surgical Procedures”[Title/Abstract] OR “Embryoscopic Surgical Procedure”[Title/Abstract] OR “Procedure, Embryoscopic Surgical”[Title/Abstract] OR “Procedures, Embryoscopic Surgical”[Title/Abstract] OR “Surgical Procedure, Embryoscopic”[Title/Abstract] OR “Surgery, Embryoscopic”[Title/Abstract] OR “Surgical Procedures, Embryoscopic”[Title/Abstract] OR “Embryoscopic Surgery”[Title/Abstract] OR “Embryoscopic Surgeries”[Title/Abstract] OR “Surgeries, Embryoscopic”[Title/Abstract])). Filters: in the past 10 years, English.

### Study Selection

The search results of each database were gathered using the Rayyan online software (Rayyan Systems Inc., Cambridge, MA, US). 16 After the exclusion of duplicates, the publications were independently screened by title and abstract by J.C.S. and J.R.B. regarding the eligibility criteria. After both authors finished screening the search results, the study selection was compared, and disagreements were resolved by consensus. The remaining studies were further independently screened by full-text reading by J.C.S. and J.R.B., and those that met the eligibility criteria were assessed for data extraction. The exclusion criteria weere wrong type of publication, wrong population, studies with animal models, or experimental studies.

### Data Extraction

Data were extracted using an online data extraction form specifically designed for this review and piloted prior to the data collection. J.C.S. performed data extraction, and all information was checked in a second data extraction performed by J.C.S and J.R.B. The form included: study design, total of participants, number of patients randomized per group, number of patients excluded after randomization, maternal age, parity (nulliparous/parous), side of the lesion (left/right), liver herniation, LHR, o/e LHR, gestational age (GA) at FETO, GA at balloon removal, maternal death, perinatal death, infant death until 6 months of age, maternal admission to intensive care unit (ICU), maternal blood transfusion, chorioamnionitis, birth before 37 weeks, preterm premature rupture of membranes (PPROM), total of days of stay in the neonatal ICU, oxygen use at infant discharge, and pulmonary hypertension (as defined by the included studies).

### Risk of Bias

The assessment of the risk of bias was performed for every reported outcome and for individual studies using the Cochrane RoB 2 tool. 9 17 Both reviewers performed the risk of bias assessment, and any disagreement was resolved by consensus. This tool consists of five domains: 1) risk of bias arising from the randomization process; 2) risk of bias due to deviations from the intended interventions (effect of assignment to intervention); 3) missing outcome data; 4) risk of bias in the measurement of the outcome; and 5) risk of bias in the selection of the reported result. Every domain consists of questions approaching aspects of the publication which could indicate bias. The answers to these questions characterize the domains as “low risk of bias,” “high risk of bias” or “some concerns.” Finally, the study receives an overall classification of risk of bias.

### Measures of Effect

When applicable, continuous outcomes were measured by mean differences and 95% confidence intervals (95%CIs); dichotomous outcomes were measured by risk ratio/relative risk (RR) and 95%CIs.

### Data Synthesis

Elements of the Patient, Intervention, Comparison, Outcomes (PICO) strategy, study design features of the included publications and numerical data were described in tables. Data were summarized in a summary of findings table. The GRADE approach was used to assess the quality of the evidence for all outcomes: a “high certainty” label is given when there is confidence that the true effect is close to that of the estimate of the effect; a “moderate certainty” label is given when the true effect is probably close to the estimate of the effect, but it is possible that it is substantially different; a “low certainty” label is given when the true effect may be very different from the estimate of the effect; and a “very low certainty” label is given when the true effect is probably substantially different from the estimate of the effect. 10 11 12 13 14

## Results

### Study Selection

**Fig. 1:**
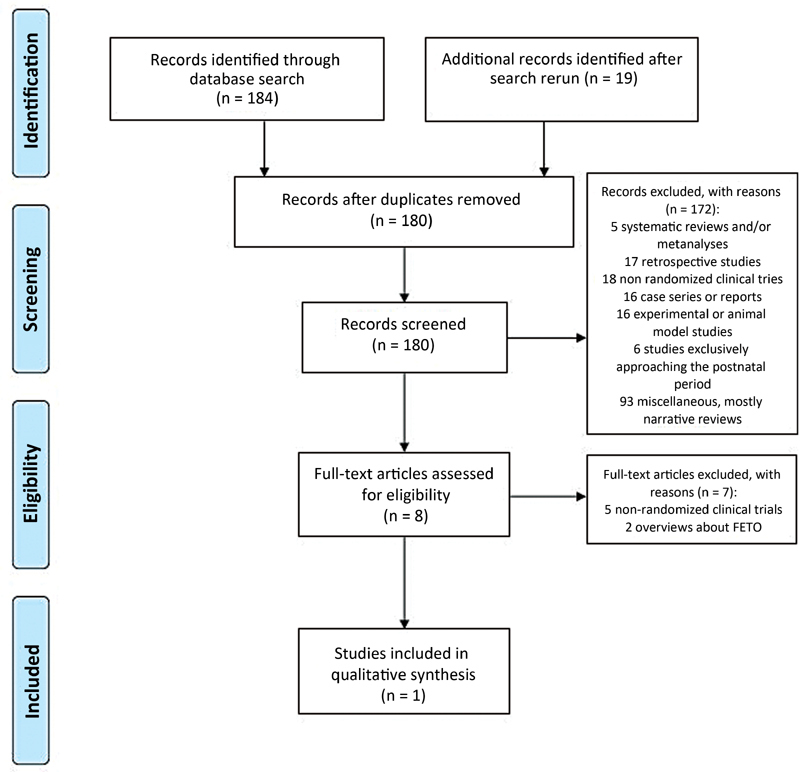
Flowchart of the screening and selection process of the rapid review. The reasons for the exclusion of studies are listed in the boxes. **Source:** Moher et al. 15

Through the searches in the MEDLINE, PMC, EMBASE and CENTRAL databases, we retrieved 203 publications which were exported to the Rayyan software. Duplicates (23 studies) were excluded prior to the first step of screening. Initially, both authors independently screened all the 180 publications by title and abstract. There were 19 discrepant evaluations between the authors, which were resolved by consensus, and a final list of eight potentially-eligible studies was developed. The reasons for exclusion were studies with animal models or experimental studies, wrong type of publication, wrong study design, or wrong population. The next step in the study selection was reading the full texts of the remaining articles. They were independently screened by full text by both authors, and one study 18 that met the eligibility criteria was identified, and is described in the next sections. The publications excluded in the first step comprised 5 systematic reviews and/or metanalyses, 17 retrospective studies, 18 non-randomized clinical trials, 16 case reports or series, 16 experimental studies or animal model studies, 6 studies exclusively approaching the postnatal period, and 93 miscellaneous studies, which were mostly narrative reviews. Fig. 1 shows the PRISMA flowchart of the selection of studies.

### Study Characteristics

One study 18 met the inclusion criteria for the present review. This single-center study evaluated pregnant women whose fetuses presented isolated CDH (no other malformation apart from CDH), normal karyotype, LHR < 1.0, and at least one third of the liver herniated into the thoracic cavity as estimated by ultrasound. After applying the inclusion criteria and obtaining informed consent, the authors randomized 41 women into 2 groups (expectant management and FETO) with a computer-generated randomization scheme at a 1:1 ratio. After randomization, 20 women were assigned to the FETO group (also referred to as the intervention group), and 21, to the expectant management group (also referred to as the control group or controls). One patient in the FETO group and two in the expectant management group declined the assigned intervention after randomization, and were excluded from the study. Chart 1 highlights the main aspects of the study. 18 At 26 to 30 weeks of gestational age, FETO was performed, and the authors planned elective balloon removal by ex utero intrapartum treatment (EXIT) at 38 weeks, which was not performed in all cases due to PPROM or preterm labor. The newborns of both groups underwent the same perinatal management, and were followed up until 6 months of age. The authors performed intention-to-treat and received-treatment analyses.

**Chart 1:** Characteristics of the study reviewed

	Ruano et al. 18 (2012)
**Study design**	Randomized controlled trial
**PICO: patient**	Pregnant women whose fetuses presented isolated Congenital diaphragmatic hernia (with no other malformation), normal karyotype, lung-to-head ratio < 1.0, and at least one third of the liver herniated into the thoracic cavity as estimated by ultrasound.
**PICO: intervention**	Fetal endoscopic tracheal occlusion at 26 to 30 weeks of gestational age and programmed balloon removal at 38 weeks
**PICO: comparison**	Antenatal expectant management
**PICO: outcomes**	Primary outcome: survival until 6 months of age. Additional outcomes: maternal complications, severe postnatal pulmonary hypertension and length of time until surgical repair of the diaphragmatic defect. ^a^

Note: ^a^ This outcome was not included in this review.

### Study Results

In the study, 18 the mean maternal age was 29.5 ± 6.6 years in the FETO group, and 30.3 ± 6.4 years in the control group ( *p* = 0.85). In the FETO group, 60% of the women were nulliparous; in the control group, 57.1% were nulliparous ( *p* = 0.76). Parous women constituted 40% of the intervention group and 42.9% of the expectant management group ( *p* = 0.76).

In the intention-to-treat analysis, the groups did not differ in terms of the side of the diaphragmatic defect (left-sided CDH in 75% of the intervention group and in 71.4% of the control group; *p* = 0.99), LHR (0.8 ± 0.11 in the FETO group and 0.79 ± 0.10 in the expectant management group), o/e LHR (0.18 ± 0.02 in the FETO group and 0.17 ± 0.06 in the control group), neither regarding other characteristics. Fetal endoscopic tracheal occlusion was successfully performed in all cases between 26 and 30 weeks of gestation.

The obstetric outcomes were detailed, and they included mean gestational age at delivery, preterm delivery (before 37 weeks of age), and PPROM. The mean gestational age at delivery was of 35.6 ± 2.4 weeks in the intervention group (range: 31 to 38 weeks), and of 37.4 ± 1.9 weeks in the expectant management group (range: 33 to 40 weeks), with *p* < 0.01. Preterm premature rupture of membranes occurred in 35.5% of the cases in the FETO group, and in 23.8% of the controls ( *p* = 0.51).

Maternal morbidity and mortality were reported for both groups: there were no cases of maternal death or maternal blood transfusion in the study, and there was 1 (5%) case of chorioamnionitis in the intervention group, which was diagnosed after PPROM. There were no cases of chorioamnionitis among the controls.

Neonatal and infant outcomes were presented as intention-to-treat and received-treatment analyses. In the intention-to-treat analysis, 50% of the newborns who had undergone FETO and 5.8% of those in the control group survived until 6 months of age ( *p* < 0.01; RR: 10.5; 95%CI: 1.5–74.7). Severe postnatal pulmonary hypertension was defined as a pre- to postductal saturation difference of more than 10% associated with echocardiography confirmation, and was found in 50% of the newborns in the intervention group and in 85.7% of the controls ( *p* = 0.02; RR: 0.6; 95%CI: 0.4–0.9).

Ruano et al. 18 did not report maternal admission to ICU, neonatal ICU length of stay, perinatal mortality, and oxygen use at discharge.

All reported outcomes and their respective RRs and 95%CIs are detailed in Chart 2 .

**Chart 2:** Summary of findings: Ruano et al. 18 (2012)

Outcomes ^a^	Number of participants (studies)	Certainty of the evidence (GRADE)	*Risk ratio (95% confidence interval)	Anticipated absolute effects	
				Risk with no intrauterine intervention	Difference in risk with fetal endoscopic tracheal occlusion
Infant survival until 6 months	41 (1 randomized controlled trial)	Moderate ^b^	10.50 (1.47–74.71)	48 per 1,000	452 more per 1,000 (22 more to 3,510 more)
Severe postnatal pulmonary hypertension	41 (1 randomized controlled trial)	Moderate	0.58 (0.36–0.93)	857 per 1,000	360 fewer per 1,000 (549 fewer to 60 fewer)
Chorioamnionitis	41 (1 randomized controlled trial)	Low ^c^	Not estimable	0 per 1,000	0 per 1,000
Preterm birth	41 (1 randomized controlled trial)	Moderate	1.75 (0.78–3.91)	286 per 1,000	214 more per 1,000 (63 fewer to 831 more)
Preterm premature rupture of membranes	41 (1 randomized controlled trial)	Low ^c^	1.47 (0.55–3.88)	238 per 1,000	112 more per 1,000 (107 fewer to 686 more)

Abbreviation: GRADE, Grading of Recommendation, Assessment, Development and Evaluation (GRADE) guidelines.

Source: Ruano et al. 18

Note: *The risk in the intervention group (and its 95% confidence interval) is based on the assumed risk in the control group and the relative effect of the intervention (and its 95% confidence interval).

### Explanations

- Chart 2 shows outcomes that were reported in at least one of the groups. Chorioamnionitis was only reported in the intervention group, so the RR cannot be estimated. We intended to analyze maternal admission to ICU, neonatal ICU length of stay, perinatal mortality, and oxygen use at discharge, but these outcomes were not included in the study by Ruano et al. 18
- The range of the 95%CI is wide, leading to imprecision.

One patient in the FETO group presented chorioamnionitis, and the authors report this happened after PPROM. Although PPROM was measured in both groups, there is no description of the diagnostic procedures used, and if these procedures were standardized between groups. Furthermore, it is not clear whether chorioamnionitis was clinical and/or histopathological, nor if this affected patient had preterm delivery, which could be a confounding factor if chorioamnionitis was limited to the histological findings. This could lead to information bias.

### Risk of Bias

The evaluation of the risk of bias was performed by both reviewers using the RoB2 tool for outcomes that were present in both groups (newborn survival until 6 months of age, severe postnatal pulmonary hypertension, PPROM, delivery before 37 weeks of gestation) and for an overall assessment of the study 18 in question. The sources used for this evaluation were the published article 18 and the information available at Clinical Trials' webpage. 19

**Fig. 2:**
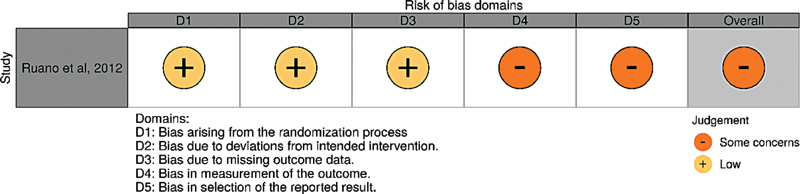
Risk of bias of each individual RoB 2 domain and overall evaluation. Due to some concerns about domains 4 and 5, the overall evaluation of the study is some concerns of risk of bias. **Source:** Ruano et al. 18

Preterm delivery was at a low risk of bias; PPROM, newborn survival until 6 months of age and severe postnatal pulmonary hypertension raised some concerns; PPROM, because there was no description of how this diagnosis was established, which could lead to detection bias, and newborn survival until 6 months of age and severe postnatal pulmonary hypertension, due to possible reporting bias, as neither of these were intended to be described in the Clinical Trials protocol. Therefore, an analysis of the study indicated some concerns of bias. Figure 2 shows the overall evaluation using the Cochrane risk-of-bias visualization (robvis) tool. 17

### Quality of Evidence

The quality of the evidence was assessed according to GRADE definitions. Moderate certainty was considered for preterm birth, newborn survival until 6 months and severe postnatal pulmonary hypertension. The reasons for this classification were broad 95%CIs, single study, and small sample. Chorioamnionitis and PPROM were considered of low certainty because there was no description of the diagnostic tests used to identify the cases of PPROM or chorioamnionitis, which could lead to information bias. Moreover, it is not clear whether chorioamnionitis followed a preterm delivery and this is another possible limitation.

## Discussion

### Summary of the Main Results

The collected evidence suggests that FETO may be an effective intervention to reduce severe postnatal pulmonary hypertension and to enhance survival rates until 6 months of age. It is not possible to make assumptions about the role of FETO in the maternal or obstetric outcomes. This uncertainty is explained by the small sample of the only study 18 that met the inclusion criteria. Moreover, in the included study, 18 some of the outcomes we planned to analyze were not described.

### Quality of Evidence

The analyzed evidence was evaluated as of moderate to low certainty according to GRADE definitions, which means that, in the best-case scenario, the estimate of the effect is probably close to the true effect of the intervention, although it is possible that the true effect may be substantially different. The small sample of the only RCT 18 that met the inclusion criteria for the present review might clarify why some of the outcomes were not observed in both groups. Additionally, the high RR and broad 95%CIs suggest that future research might revise these estimates.

### Implications for Practice and Research

Fetal endoscopic tracheal occlusion was introduced in the clinical practice as a percutaneous procedure in 2004. 5 Ever since, various attempts to improve the technique have been made, and currently there is a better understanding of the pathophysiology of the disease before and after FETO. 20 21 22 23 24 25 26 27 28 29 30 31 32 33

Although CDH has been extensively studied, there are many gaps in the current knowledge, especially regarding perinatal outcomes. The clinical implications of these are challenges during patient counselling and suitable antenatal management, 34 which can only be resolved when large and well-conducted RCTs are available. Meanwhile, some authors 4 34 35 36 advocate that FETO should preferably be considered in the setting of clinical trials. In our opinion, even though the evidence is limited, the potential improvements in postnatal survival and reduction in pulmonary hypertension may justify performing FETO in the clinical practice.

### Strengths and Limitations

The reliability of this rapid review is endorsed by the strict adherence to the proposed methods: registration of the protocol prior to conducting the searches, thorough use of the Cochrane Rapid Reviews Methods, 8 evaluation of the risk of bias with a recommended tool (RoB 2), 9 17 assessment of the quality of the evidence with worldwide accepted guidelines (GRADE), 13 14 15 16 17 and development of the final report according to the PRISMA statement. 15 Every measured outcome is clinically relevant, although the evidence is limited. Some limitations for the present study are expected. Congenital diaphragmatic hernia is a rare condition, which restricts the availability of data. Assuming that the searches were conducted in the main databases of the medical literature, it is unlikely that RCTs meeting our inclusion criteria were missed due to the requirements of a rapid review. The present review could not provide strong evidence for any outcome or subgroup analyses due to the small sample of the study 18 reviewed – these important issues of medical research might be overcome by future publications.
